# Access to surgical care in Ethiopia: a cross-sectional retrospective data review

**DOI:** 10.1186/s12913-022-08357-9

**Published:** 2022-07-30

**Authors:** Berhane Redae Meshesha, Manuel Kassaye Sibhatu, Hassen Mohammed Beshir, Wuletaw Chane Zewude, Desalegn Bekele Taye, Edlawit Mesfin Getachew, Kassa Haile Merga, Tsegaye Hailu Kumssa, Endawoke Amsalu Alemayue, Akililu Alemu Ashuro, Mulatu Biru Shagre, Senedu Bekele Gebreegziabher

**Affiliations:** 1 Ministry of Health of Ethiopia, Addis Ababa, Ethiopia; 2 Jhpiego Ethiopia, Johns Hopkins University Affiliate, Addis Ababa, Ethiopia; 3 St. Paul’s Hospital Millennium Medical College (SPHMMC), Addis Ababa, Ethiopia; 4 College of Medicine and Health Science, Addis Ababa University (AAU), Addis Ababa, Ethiopia; 5 Armauer Hansen Research Institute (AHRI), Addis Ababa, Ethiopia; 6 Faculty of Medicine, Department of Health Sciences, Child and Family Health, Lund University, Lund, Sweden

**Keywords:** Surgical care, Access, Cross-sectional, Retrospective data, Ethiopia

## Abstract

**Background:**

Access to emergency and essential surgical care is still unmet and accessibility is disproportionately inequitable in Ethiopia and other low-and middle-income countries. The aim of this study was to assess surgical care access in terms of capability, capacity, and timeliness of care in different levels of health care in Ethiopia.

**Methods:**

A cross-sectional study with retrospective data review was conducted in 172 health facilities from December 30, 2020 to June 10, 2021. Descriptive statistics such as median with interquartile range and proportion were computed using STATA Version 15 statistical software.

**Results:**

Within a 90-day interval of the study period, 69,717 major and minor surgeries, and 33,052 bellwether procedures were performed, and major surgeries accounted for 58% of the surgeries. About 1.6%, 23.56%, 25.34%, and 32.2% of both major and minor, and 3.1%, 12.8%, 27.6%, and 45.3% of bellwether procedures were performed in health center OR blocks, primary, general, and specialized hospitals, respectively. Private hospitals performed 17.33% of major and minor and 11.2% of bellwether procedures for the period. The average pre-admission waiting time for surgical patients in primary, general, and specialized hospitals was 9.68, 37.6, and 35.9 days, respectively, whereas, in private hospitals, the average pre-admission waiting time was 1.42 days. On average, surgical patients traveled 5 Hrs, 11 Hrs, 28.4 Hrs, and 21.3 Hrs to access surgical services in primary, general, specialized, and private hospitals, respectively. The surgical workforce to the population served ratio was 7.5, 1.15, and 1.31/100.000 population in primary, specialized and general hospitals, respectively.

**Conclusion:**

Most surgical procedures were performed in specialized hospitals, indicating that there is a burden in these health facilities. The pre-admission waiting time for surgical patients was long in higher-level public hospitals. Surgical patients traveled a long distance to access surgical service in higher level hospitals. The ratio of surgical workforce per 100,000 population served was low in all levels of public health facilities in general, and in higher level hospitals in particular. Efforts should therefore be made to strengthen all levels of the health system and improve surgical care access in terms of capacity, capability, and timeliness in the country.

## Background

Equitable access to surgical care service is still unreachable for billions of people. According to the Lancet Commission on Global Surgery (LCoGS), about five billion people lack access to timely, safe and affordable surgical and anesthesia care services globally [1]. However, the inaccessibility of surgical care disproportionately high in low-and middle-income countries (LMICs), where nine of ten people cannot access basic surgical care. Of the 313 million surgical procedures undertaken globally per year, only 6% were performed in the poorest countries, where over a third of the world’s population lives. The lion share of unmet surgical needs appear in sub-Saharan Africa and South Asia [1]. Regarding the type of procedures, 80% of the practices are indicated to be elective, whereas, in sub-Saharan Africa major proportion of the practice is on emergency and essential surgeries. This requires instantaneous attention [2]. Improving access to safe and affordable emergency and essential surgical and anesthesia care reduces premature death and disability, and also boosts welfare, economic productivity, capacity, and freedoms, contributing to long-term development [1].

The World Health Organization (WHO) Global Initiative for Emergency and Essential Surgical Care launched in 2005, galvanized global commitment for strengthening access to Emergency and Essential Surgical Care (EESC) in LMICs through successful advocacy efforts for the inclusion of EESC as an integral component of the Universal Health Coverage (UHC) packages. In 2015, the World Health Assembly (WHA) Resolution on EESC had motivated countries to prioritize surgical and anesthesia care in their national surgical plans and develop a national surgical plan [3, 4].

Measuring surgical care in terms of capability, capacity, timeliness, safety, and affordability is essential to access the services. In line with this, the LCoGS put forth the following targets to be achieved by 2030. The targets include 80 percent coverage of essential surgical and anesthesia services per country; at least 20 surgical, anesthesia, and obstetric physicians per 100,000 population; 5,000 procedures annually per 100,000 population, and 100 percent protection against catastrophic expenditure from out-of-pocket payments for surgical and anesthesia care [1].

In Ethiopia, former study findings showed a substantial unmet need for surgical care in the country. For instance, in 2015 the surgical volume and specialist surgical workforce per 100,000 population were 43/100,000 population and 0.35 surgeons/100,000 population, respectively [5]. In alignment with global commitment, the Ministry of Health (MOH)-Ethiopia launched Saving Lives through Safe Surgery I (*SaLTS I*) Strategic Plan 2016–2020 as the national flagship initiative. The *SaLTS* initiative was designed to improve access to safe surgical care at all levels of the Ethiopian health care delivery system with special emphasis in expanding EESC service in the primary level healthcare units (PHCUs) [6, 7].

Ethiopian health care operates on the basis of a three-tier healthcare delivery system. The first level comprises the PHCUs, which include health posts, health centers, and primary hospitals, while levels two and three comprise general hospitals and specialized hospitals including teaching hospitals, respectively [8]. The private for-profit sectors are supplementing the health service coverage at various levels of the healthcare system [9]. However, after the *SaLTS* initiative has been launched, there is no adequate information about surgical care access status across levels of the health system in Ethiopia. Understanding the surgical care access status will be a springboard for the subsequent planning and inform the strategies to achieve universal access to surgical care in the nation. The aim of this study was to assess the status of surgical care access in terms of capability, capacity, and timeliness in different levels of health care including public and private health care facilities in Ethiopia.

## Methods

### Study design and setting

Health facility based cross-sectional study with retrospective data review was conducted in public and private health facilities of Ethiopia from December 30, 2020 to June 10, 2021. Ethiopia is an east African country with estimated population of more than 117 million people in 2021 [10]. A total of 282 government health facilities and 45 private hospitals were providing surgical care in the country during the study period.

### Sampling procedure and sample size

A multi-stage stratified random sampling method was used to select study sites (public and private health care facilities). First, lists of all health care facilities providing surgical care were obtained from MOH of Ethiopia’s District Health Information System 2 (DHIS 2) report. Accordingly, 282 government hospitals (26 specialized hospitals, 75 general hospitals, and 181 primary hospitals) were providing EESC during the study period. The required sample size for the study was estimated using a single population proportion formula with a 95% level of confidence, a 5% margin of error, and an assumed proportion of surgical care accessibility (*P* = 0.5); therefore, the sample size was estimated to be 163 public hospitals. As we used a stratified sampling method, the sample size for each stratum of primary hospitals (n_p_), general hospitals (n_g_), and specialized hospitals (n_s_) was calculated using the proportional allocation method and it was 105, 43, and 15 hospitals, respectively. In addition, to assess the status of surgical care in the private health sector, private health facilities providing safe surgical care services were included. According to DHSI 2 report, 45 private health facilities were providing surgical care. Thus, the sample size for private hospitals was estimated to be 40. This makes a total of 203 study sites. Each hospital from each stratum was selected by a simple lottery method. However, due to internal conflict and limited transport accessibility in some parts of Ethiopia, we could not be able to access all health facilities that were selected randomly, and we decided to replace some health facilities which were convenient assuming that hospitals in the same strata are homogeneous. A total of 172 sampled health facilities were evaluated in the study, which hold 84.7% of the estimated sample size.

### Data sources and collection

Routine DHIS 2 surgical care services database and pre-admission and admission register were reviewed using the Harvard Program in Global Surgery and Social Change and the WHO Surgical Assessment Tool [11], which was adopted in the context of Ethiopia. Thirteen trained data collectors reviewed the data from December 30, 2020-June 10, 2021. Data collectors were trained about the entire process of data collection including quality control measures such as: completeness, correctness, consistency, and synchronizing and archiving data with RedCap. The data we reviewed includes data dating back to September 2020 to May 2021; specifically, for total numbers of surgical procedures with the intention to capture the volume of procedures done in the past 90 days prior to data collection, therefore, this data reflects the volume of procedures done in a 90-day interval of the time from September 2020 to May 2021. Precaution measures including wearing a face mask, using hand sanitizers, and physical distancing were implemented to prevent Coronavirus disease 2019 (COVID-19) transmission during data collection.

### Quality assurance

To ensure the quality of data, the data were cleaned and checked for completeness, correctness, and consistency. Regular supervision and follow-up were made throughout the data collection period.

### Operational definition of variables

Surgical care: provision of perioperative and operative management for surgical conditions.

Surgical volume: number of minor and major surgical procedures performed during the study period.

Major surgery: surgeries that require general or regional anesthesia, involve opening great body cavities, have risk of severe hemorrhage, put the patient’s life at risk and needs postoperative care and require special anatomical knowledge, manipulative skills,and specific equipment.

Minor Surgery: surgeries in which short surgical techniques are applied on superficial tissues, usually with local anesthesia and minimal complications that usually do not require postoperative resuscitation and need minimal equipment.

A Bellwether procedure: any procedure involving laparotomy, cesarean section, or treatment of an open long bone fracture.

Physical access for surgical care: Surgical health facilities that can be accessed within two hours of travel.

Surgical referrals out: number of patients referred out of the hospitals/health center operation room (OR) blocks for surgical services after an on-site assessment by a medical professional in the reporting period.

Surgical workforce: total number of available surgical workforces including Surgeons (General, neurosurgeons, and orthopedic surgeons), Anesthesiologists or anesthesia care providers, Obstetrician-gynecologist, Integrated Emergency Surgical Officers (IESO), and Nurse Anesthetists.

### Data management and analysis

The reviewed data were cleaned, checked for consistency, and entered into the Redcap database, and the data collectors archived cleaned data on a regular basis, every week. Then the cleaned data were exported into STATA Version 15 statistical software package for statistical analysis.

Descriptive statistics: frequency, proportion, and median with interquartile range (IQR) were computed. As the data were skewed, non-parametric tests (Mann–Whitney/Kruskal–Wallis) were employed to compare the median number of surgical volumes performed by health care facilities level. Kruskal–Wallis test was used for comparing the surgical volume performed among public specialized hospitals, public general hospitals, and public primary hospitals, and the Mann–Whitney test was used for comparing the surgical volume between public and private health facilities. A *p*-value of < 0.05 was considered statistically significant.

### Ethical consideration

The study was approved by Armauer Hansen Research Institute (AHRI) ethical review board in Addis Ababa, Ethiopia. Additionally, the Ethiopia MOH issued a letter of support to conduct data review at the selected health facilities. Additionally, letters of support and permissions were obtained from the local health offices authorities to conduct data review at the selected health facilities.

## Results

### Evaluated health facilities

A total of 172 health facilities were included in the study. Of which, 9 (5.2%), 77 (44.8%), 38 (22.1%), 16 (9.3%), and 32 (18.6%) were health center OR blocks, primary hospitals, general hospitals, specialized hospitals, and private hospitals, respectively.

### Surgeries performed by health care facilities

Within a 90-day interval of the study period, from September 2020 through May 2021 a total of 69,717 major and minor surgical procedures were performed. Of which were 40, 202 (58%) major surgeries and 29,515 (42%) minor surgeries. With regard to the share of facilities, 40.7%, 27%, and 13.3% major procedures were performed at specialized, general, and primary hospitals, respectively. On the other hand, 20.5%, 23.1%, and 37.6% minor surgical procedures were undertaken in specialized, general, and primary hospitals, respectively. Private hospitals performed 18% of major and 16.4% of minor surgical procedures. One percent of major surgery (elective cesarean section) and 2.4% of minor surgeries were performed in health centers OR blocks (Table 1).

**Table 1:** Major and minor surgeries the evaluated health facilities performed, September 2020—May 2021, Ethiopia

Level of health care facilities	Surgical procedures performed in a 90-day interval		
	Major surgical procedures Number (%)	Minor surgical procedures Number (%)	Total major and minor surgical procedures Number (%)
Health centre OR block	403 (1.0)	718 (2.4)	1,121 (1.6)
Public primary hospital	5,341 (13.3)	11,084 (37.6)	16,425 (23.6)
Public general hospital	10,855 (27.0)	6,810 (23.1)	17,665 (25.3)
Public specialized hospital	16,364 (40.7)	6,056 (20.5)	22,420 (32.2)
Private hospital	7,239 (18.0)	4,847 (16.4)	12, 086 (17.3)
Total	40, 202 (100%)	29, 515 (100%)	69, 717 (100%)

This table shows the number of major and minor surgical procedures that were performed in a 90-day interval from September 2020 to May 2021, disaggregated by level of health care facilities, Ethiopia

The study also showed that a total of 33,052 bellwether procedures were performed during the period. Public specialized hospitals performed 46.3% of Caesarean section, 34% of Laparotomies, and 49.4% of Open fracture managements. Private hospitals performed 11.2% of the total bellwether procedures including 33.4% of open fracture management, 21.3% of laparotomy, and 8% of caesarean section. About 12.8% and 3.1% of the bellwether procedures were performed at PHCUs (public primary hospitals and health center OR blocks), respectively (Table 2).

**Table 2:** Bellwether procedures the evaluated health facilities performed, September 2020 to May 2021, Ethiopia

Level of health care facilities	^a^Bellwether surgical procedures performed in a 90-day interval			
	Caesarean section N (%)	Laparotomy N (%)	Open fracture management N (%)	Total bellwether surgical procedures Number (%)
Health centre OR blocks	1,037 (3.8)	1 (0.0)	0 (0)	1,038 (3.1)
Primary hospital	3,770 (13.8)	444 (13.4)	14 (0.6)	4,228 (12.8)
General hospital	7,706 (28.1)	1,036 (31.3)	392 (16.6)	9,134 (27.6)
Specialized hospital	12,673 (46.3)	1,128 (34.0)	1,162 (49.4)	14,963(45.3)
Private hospital	2,198 (8.0)	706 (21.3)	785 (33.4)	3,689 (11.2)
Total	27,384	3,315	2,353	33,052 (100)

This table shows the number of Bellwether surgical procedures that were performed in a 90-day interval from September 2020 to May 2021, disaggregated by level of health care facilities, Ethiopia

^a^Bellwether surgical procedures are subsets of major surgical procedures

Comparing the median number of Caesarean section performed in public health facilities versus private hospitals, the median number of Caesarean section procedures performed in public health facilities was significantly different from the private hospitals; 67 (IQR:37–189) versus 48 (IQR: 24–81), respectively (Mann–Whitney test, *P* = 0.02). The median number of major surgical procedures that were performed in private hospitals was higher as compared to the number of procedures performed in public health facilities; 118 (IQR: 60–287) versus 93 (IQR: 36–214) (Mann–Whitney test, *P* = 0.16) (Table 3).

**Table 3:** Comparison of surgical volume in public and private health facilities, September 2020-May 2021, Ethiopia

Surgical procedures	Number of health facilities reported surgical procedures		Surgical procedures performed in public health facilities in a 90-day interval		Surgical procedures performed in private health facilities a 90-day interval		*P*-value
	Public	Private	Median	(IQR)	Median	(IQR)	
Major surgical procedures	136	32	93	(36–214)	118	(60–287)	0.16
Minor surgical procedures	135	31	109	(51–241)	80	(42–177)	0.33
^a^Caesarean section	138	32	67	(37–189)	48	(24–81)	0.02*

^a^Caesarean section is a subset of major surgical procedures

The overall median number of major surgical procedures that were performed in the surveyed public health facilities was 105 (IQR: 40–225). The median number of major surgical procedures performed in specialized hospitals, general hospitals, and PHCUs were 1028, 185, and 46, respectively (Kruskal–Wallis test; *p* < 0.001). The median number of Caesarean sections the surveyed public health facilities performed was 105 (IQR: 50–234). Specialized hospitals, general hospitals, and PHCUs reported 373, 165, and 46 median number of Caesarean section, respectively during the study period (Kruskal–Wallis test; *p* < 0.001) (Table 4).

**Table 4:** Surgical procedures public health facilities performed, September 2020 to May 2021, Ethiopia

Public health care facilities level	Number of public health facilities reported major surgical procedures	Major surgical procedures performed by public health facilities within a 90-day interval			Number of public health facilities reported minor surgical procedures	Minor surgical procedures performed by public health facilities within a 90-day interval			Number of public health facilities reported Caesarean section	Caesarean section performed by public health facilities within a 90-day interval		
		Media	IQR	*P*-value		Median	IQR	*P*-value		Median	IQR	*P*-value
^a^Primary health care units	84	46	(22- 86)	< 0.001*	83	94	(46–219)	< 0.01*	186	46	(27–69)	< 0.001*
General hospitals	37	185	(21–349)		37	115	(69–270)		15	165	(68–227)	
Specialized hospitals	15	1,028	(715–1450)		15	193	(127–857)		16	373	(297–560)	

Using non-parametric Kruskal–Wallis test to compare three or more groups, this table shows the comparison of the median number of minor and major surgical procedures, and Caesarean section among public health facilities, in a 90 day interval, from September 2020 to May 2021, Ethiopia

^a^Primary hospitals and health center OR blocks

### Surgical volume to population ratio

Surgical volume to population ratio showed that on average, 289 surgical procedures were performed per 100,000 population in PHCUs within a 90-day interval of the study period. In specialized hospitals, the surgical volume to population ratio was found to be 33/100,000 population during the same period (Table 5).

**Table 5:** Surgical volume to population ratio, September 2020 to May 2021, Ethiopia

Level of health care facilities	Number of health care facilities evaluated	Surgical volume performed in a 90-day interval of the study period	^a^Catchment population served	Surgical volume to population ratio /100,000 population/a quarter or three months
Health center OR blocks	9	1,121	360 000	311/100 000
Public primary hospital	77	16,425	6 160 000	267/100 000
Public general hospital	38	17,665	47 500 000	37/100 000
Public specialized hospital	16	22,420	68 000 000	33/100 000

This table shows surgical volume to population ratio in a 90-day interval, from September 2020 to May 2021, disaggregated by level of health care facilities, Ethiopia

Private hospitals do not have a clearly defined catchment population

^a^Source of data for catchment population is the Ethiopian Heath sector transformation plan I [8]

Average surgical volume to population ratio of PHCUs = 289/100,000 population

### Pre-admission waiting time for patients who need essential surgical care

The recorded average pre-admission waiting time for patients, who need essential surgical care in primary, general, and specialized hospitals was 9.68, 37.6, and 35.9 days, respectively. In private hospitals, the average pre-admission waiting time recorded for patients who need essential surgical care was 1.42 days. There was no essential surgical service in the health center OR blocks.

Data reviewed from the hospital pre-admission or admission register showed the average time that surgical patients needed to get to access surgical services in public specialized hospitals and private hospitals was 28 Hours (Hrs) and 21 Hrs, respectively. The time length required to get access to surgical care in primary hospitals was exceeded 4 hours (Table 6).

**Table 6:** Physical access to a healthcare facility for surgical care disaggregated by health facilities level, Ethiopia

Health care facilities level	Average kilometer/hours that most patients travel to access surgical services
Health centre OR blocks	9.3 km (1Hrs)
Public primary hospital	49.2 km (4.92 Hrs)
Public generalized hospital	107 km (11 Hrs)
Public specialized hospital	284.3 km (28 Hrs)
Private hospital	215 km (21 Hrs)

### Surgical referral out to other health care facilities

**Fig. 1:**
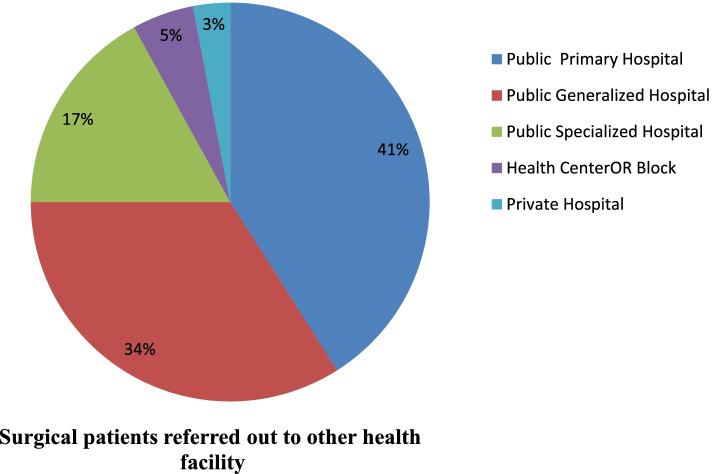
The pie chart describes the proportion of surgical patients refereed to other health care facilities in a 90-day interval, from September 2020 to May 2021, disaggregated by level of health care facilities, Ethiopia

A total of 8,584 patients were referred for surgical services in a 90-day interval. About 3956 surgical patients were referred from PHCUs to other health facilities. Of these 3540 and 416 surgical patients were referred from public primary hospitals and health center OR blocks, respectively. Public generalized and public specialized hospitals referred 2936 and 1449 surgical patients, respectively. A total of 243 surgical cases were referred out to other health facilities from private hospitals during the same period (Fig. 1).

**Fig. 2:**
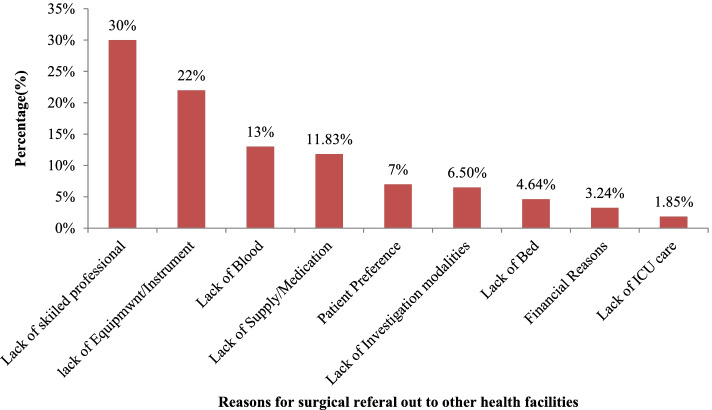
The bar graph illustrates common reasons for referral of patients for surgical intervention to other health care facilities in a 90-day interval, from September 2020 to May 2021, Ethiopia

Lack of skilled professionals and equipment/instruments constitute 30% and 22% of the most common reasons for surgical referral out, respectively. Lack of supply/medication, lack of bed, lack of blood, patient preference, lack of investigation modalities, financial reasons, and lack of Intensive Care Unit (ICU) collectively account for nearly 50% of the reasons for referral out from the surveyed health facilities (Fig. 2).

### Surgical workforce

A total of 2312 health workers were available for surgical care in the studied health facilities. About 51.14% of Surgeons (General, Neurosurgeons, and Orthopedic surgeons), 48.27% of Anesthesiologists or Anesthesia care providers, and 49.4% Obstetricians were available in public specialized hospitals. Fifty nine percent and 30.2% of Integrated Emergency Surgical Officers (IESO) were available in public primary and general hospitals, respectively (Table 7).

**Table 7:** The number of surgical workforces in a 90-day interval, September 2020 to May 2021, Ethiopia

Health care facilities level	Number of available surgical workforces in a 90-day interval					
	Surgeons (General, neurosurgeons, and orthopaedic surgeons) N (%)	Anaesthesiologists or anaesthesia care providers N (%)	Obstetrician N (%)	IESO N (%)	Nurse anaesthetists N (%)	Total N
Health center OR block	0 (0%)	19 (25.12%)	0	18 (5.44%)	2 (0.85%)	39 (1.7%)
Primary hospital	47 (7.2%)	126 (16.7%)	16 (4.79%)	194 (58.6%)	79 (33.47%)	462 (19.9%)
Generalized hospital	123 (18.7%)	157 (20.82%)	76 (22.75%)	100 (30.2%)	94 (39.8%)	550 (23.8%)
Specialized hospital	336 (51.14%)	364 (48.27%)	165 (49.4%)	10 (3.02%)	14 (5.93%)	889 (38.5%)
Private hospital	151 (22.98%)	88 (11.67%)	77 (23.05%)	9 (2.7%)	47 (19.9%)	372 (16.1%)
Total	657 (100%)	754 (100%)	334 (100%)	331 (100%)	236 (100%)	2,312 (100%)

This table shows the number of surgical workforces available in the evaluated health care facilities in a 90 day interval, from September 2020 to May 2021, disaggregated by level of health care, Ethiopia

### Surgical workforce to population ratio

The ratio of surgical workforce served was 10.8/100,000 population in health center OR blocks, 7.5/100.000 population in primary hospital, 1.15/100,000 population in general hospital and 1.31/100,000 population in specialized hospital. This makes the average surgical work force in Ethiopia to be 5.19/100,000 population (Table 8).

**Table 8:** Ratio of surgical workforce per 100,000 populations served, September 2020 to May 2021, Ethiopia

Health care facility level	Number of evaluated health facilities	Catchment population served	^a^Number of surgical workforce	Surgical workforce ratio per 100,000 population served
^a^Health centre OR block	9	360 000	39	10.8
Public primary hospital	77	6 160 000	462	7.5
Public general hospital	38	47 500 000	550	1.15
Public specialized hospital	16	68 000 000	889	1.31

This table shows the ratio of surgical workforce per 100,000 populations served in a 90-day interval of the reporting period, from September 2020 to May 2021, disaggregated by level of care, Ethiopia

^a^ The surgical workforce to population ratio for each health facility was calculated from the total number of available surgical workforces including Surgeons (General, neurosurgeons and orthopedic surgeons), Anesthesiologists or anesthesia care providers, Obstetrician-gynecologist, IESO and Nurse Anesthetists

## Discussion

In this study, more than half (58%) of the surgical procedures were major surgeries, and about 41% of which were performed in public specialized hospitals where capacity in terms of human resources, infrastructure, equipment, and supplies are relatively better. On the other hand, public primary hospitals performed a small proportion (13.3%) of major surgical procedures. This is in line with a study in three East African countries that reported low rates of major surgery at district hospitals, ranging from 50 to 450 surgical procedures per 100,000 population [12]. In Ethiopia, more than 80% of the total population lives in rural areas of the country where they get health services from the PHCUs [13, 14]. This implies that there is a substantial unmet need for major surgical services for the majority of the population in Ethiopia.

Despite the fact that only 18.6% of the study facilities were private surgical facilities, the private sector contributed to 17.3% of both minor and major surgical volume for the study period. Although, the median number of major surgical procedures was significantly high in public specialized hospitals as compared to public primary and general hospitals (Kruskal–Wallis test; *p* < 0.001), however, the median number of major surgical procedures which were performed in 32 private health facilities was higher than 136 public health facilities performed during the period. Furthermore, more than a third of open fracture management was undertaken in private hospitals. This may indicate the demand for surgical care services from private health facilities and the significant contribution of private hospitals in reducing the surgical burden. Nevertheless, the private sector in Ethiopia is relatively small (approximately 20% of the total health market share) compared to other countries in the region. For instance, 46% and 65% of all health facilities are managed by Private Sectors in the Democratic Republic of Congo and in Kenya, respectively [15]. Moreover, SaLTS I strategy has not been implemented in the private hospitals. Our finding underscores the importance of scaling up surgical care services in the private sector.

In this study, nearly half (45.3%) of the bellwether surgical procedures were performed in specialized hospitals. This is consistent with findings of a study in KwaZulu**-**Natal Province of South Africa which showed that the majority of non-obstetric bellwether operations were performed at regional and tertiary hospitals [16]. The Lancet commission recommends that all first-level hospitals should be able to perform laparotomy, cesarean delivery, and treatment of open fractures as bellwether procedures [1]. The procedures have been proposed as proxy metrics for surgical systems that are functioning at a level of complexity advanced enough to provide most other surgical procedures [16, 17]. However, in the current study, first-level health care units (primary hospitals and health center OR blocks) performed a small proportion (12.8% and 3.1%) of all the bellwether procedures, respectively. Likewise, a study from KwaZulu**-**Natal Province reported that the non-obstetric bellwether operations that were performed at district hospitals of South Africa were small to negligible, with 2.1% laparotomies and 1.8% open reduction of fractures, and this study highlighted that the imbalance performance has major implications for strategic planning around the delivery of surgical care [16].

Our study showed the surgical volume of 289 /100,000 population, 37/100,000 population, and 33/100,000 population over a 90-day interval in PHCUs, general hospitals, and specialized hospitals, respectively. This indicates that the surgical volume in Ethiopia’s public health facilities falls far below the LCoGS target of 5000 surgeries per 100,000 population/year which has been set to be achieved by the year 2030 [1]. Likewise, findings of studies from other African countries showed low surgical volume to population ratios such as Uganda (145/100,000 population) [17] and Rwanda (429/100,000 population) [18].

The study revealed that patients traveled about 28Hrs, 21Hrs, and 11Hrs to access surgical services in public specialized hospitals, private hospitals, and public generalized hospitals, respectively. This is still far from the LCoGS recommendation that patients should access a facility capable of performing the Bellwether procedure within two hours [1]. The relatively long-distance travel observed in our study may be related to the reason that majority of specialist surgical workforce is available in these health facilities. Likewise, studies in other sub-Saharan African countries showed that a significant proportion of people lack timely access to surgical care. For instance, a study conducted in Zambia showed that only 20% of the population lives within two hours of facilities providing essential and emergency care [19]. Another study from Ghana revealed that about 30% of Ghanaians don’t have access to essential surgery within two hours [20]. Long-distance was one of the major factors that affect timely access to health care services in these studies [19, 20].

On the other hand, our study showed that patients have taken less than one hour to reach the health centers OR blocks. This may be related to the reason that all health center OR blocks are located in Addis Ababa where with relatively better transport access. However, a small proportion, 2.4% of minor surgeries and 3.1% of bellwether procedures were performed in health centers OR blocks for the period. This may be related to the gap in the deployment of IESO at the health center level.

The average pre-admission waiting time for patients, who need essential surgical care, was 38 days in general hospitals and 36 days in specialized hospitals. This is longer than the findings of studies from other countries. For instance, a study from Zambia showed that the pre-admission waiting time for surgical patients, who need elective surgery, in teaching hospitals was 9 days [21]. Another study from India revealed a pre-admission waiting time of 12 days for surgical patients in teaching hospital [22]. Moreover, generalized and specialized hospitals reported longer pre-admission waiting times compared to private hospitals and public primary hospitals reported. Long pre-admission time in generalized and specialized hospitals observed in this study might be related to the high burden of surgical cases requiring more advanced surgical care. The pre-admission waiting time reported in these health facilities was also longer than the country’s target of less than 30 days [8]. Prolonged pre-admission waiting time worsens surgical outcome, pre-operative anxiety score, and depression [23, 24]. Moreover, it may also result in post-operative complications, mortality and increased hospitalization stay, and catastrophic costs [25].

This study showed that public primary hospitals referred nearly two-fifths (41%) of surgical patients. Nonetheless, higher levels of health facilities including generalized and specialized hospitals also referred significant proportion of patients who need surgical interventions. Lack of skilled professionals, lack of equipment/instrument, lack of blood, and lack of supplies or medications were the most frequent reasons for patient referrals at all levels of the health system. Likewise, a study from Tanzania showed that the lack of essential equipment, infrastructure, and human resources were significant gaps to provide EESC in first-referral health facilities [26].

Lack of skilled professionals accounts for about one-third of the reasons for surgical referral out and which may likely indicate shortages of surgical workforce in the country. Our study also showed a considerably low surgical workforce to population ratio which was about 1/100,000 population served in higher levels of health facilities. However, the surgical workforce per 100,000 population served in PHCUs (10.8/100,000 pop in Health center OR blocks and 7.5/100,000 pop in primary hospitals) was higher compared to in higher levels of health facilities. This might be related to that PHCUs are supposed to serve nearby communities and lower catchment populations [8]. Shortage of surgical workforce has an impact on the service delivery, patient satisfaction, and finical burdens on the patients [27]. Unless the country designs a strategy like surgical task shifting programs it will be unlikely to achieve the LCoGS target of 20 surgical workforces per 100,000 populations by 2030 in general and accessing the surgical care service at PHCUs level in particular.

The study also has strengths. As far as our literature review is concerned, this is the first study in Ethiopia that attempted to assess surgical care access in terms of capability, capacity, and timeliness across levels of the health care system. The study highlighted the surgical care access gap within the health system of the country; therefore, we believe that the results would inform a crucial input for the surgical care strategic plane of Ethiopia and provide a foundation for evidence-based decision-making and evidence-informed policy making. Moreover, the study may be used as baseline data for future studies.

Our study has limitations. Although we could collect data from 85 percent of sampled health facilities, because of security situations related to the existing conflict at different places of the country, the data were not collected from all sampled health facilities. Moreover, the study was conducted in the COVID-19 era when the pandemic has been affecting health services provision including surgical care. Therefore, the pandemic might have affected the performance of surgical care services, particularly the surgical volume. This study may share the inherent limitation of secondary data such as inconsistency, incompleteness, and inaccuracy; however, extensive efforts have been made by the research team to ensure the data quality.

## Conclusion

The study showed that within a 90-day interval (September 2020-May 2021), the majority of surgical procedures were performed in specialized hospitals, indicating that there is a very high burden in these health facilities. Private hospitals substantially contributed for providing the surgical care service. The study revealed long pre-admission waiting time for surgical patients in higher levels of public hospitals. The surgical workforce ratio per population served was low in all levels of public health care facilities in general, and in specialized public health facilities in particular. Substantial proportions of surgical patients were refereed from public primary, generalized, and specialized hospitals to other health care facilities, indicating the weak status of the country’s health system. Surgical patients in Ethiopia traveled a long distance to access surgical services in a higher level of health facilities.

Increasing access to surgical services and reducing delays in admission would help to increase the use of the respective services. Strengthening the capacity of primary-level health facilities may decrease the number of referrals and reduce the burden on high-level health facilities. Implementing the national surgical care strategies in private hospitals has paramount importance of the surgical care provision of the nation. Overall, efforts should therefore be made to strengthen all levels of the health system and improve surgical care access in terms of capacity, capability, and timeliness in the country.

## Data Availability

The datasets used and/or analyzed during the current study are available from the corresponding author on reasonable request.

## Abbreviations

AAU: Addis Ababa University
AHRI: Armauer Hansen Research Institute
COVID-19: Coronavirus disease 2019
C/S: Caesarean Section
DHIS 2: District Health Information System 2
EESC: Emergency and Essential Surgical Care
GIEESC: Global Initiative for Emergency and Essential Surgical Care
HMIS: Health Management Information System
Hrs: Hours
IESO: Integrated Emergency Surgical Officers
ICU: Intensive Care Unit
IQR: Interquartile range
LCoGS: Lancet Commission on Global Surgery
LMICs: Low-and middle-income countries
MOH: Ministry of Health
OR: Operation room
PHCUs: Primary level healthcare units
*SaLTS*: Saving Lives through Safe Surgery
SPHMMC: St. Paul’s Hospital Millennium Medical College
UHC: Universal Health Coverage
WHA: World Health Assembly
WHO: World Health Organization
